# Penile Fracture: Experience from a Third World Country

**DOI:** 10.1155/2013/708362

**Published:** 2013-07-16

**Authors:** Rajandeep Singh Bali, Arshad Rashid, Majid Mushtaque, Shakeeb Nabi, Sajad Ahmad Thakur, Rouf Ahmad Bhat

**Affiliations:** ^1^Department of General Surgery, Maulana Azad Medical College, New Delhi 110002, India; ^2^Maharishi Markendeshwar Institute of Medical Sciences and Research, D-22, Residential Complex, MMU Complex, Mullana, Ambala 133203, India; ^3^Minimal Access Surgery, Maulana Azad Medical College, New Delhi 110002, India; ^4^Department of General Surgery, Government Medical College, Srinagar 190010, India

## Abstract

*Aim*. To ascertain the clinical presentation, commonest age group affected, and treatment of patients diagnosed to have penis fracture. *Materials and Methods*. We performed a retrospective study carried at a tertiary care hospital from January 2005 to January 2011. All the 36 patients diagnosed to have penile fracture were enrolled in the study group. The diagnosis was made based on the clinical findings in the patients. All, except two patients, were managed by a standard surgical procedure, same for all the patients, on the day of presentation to the hospital. All the data pertaining to the presentation, management, and followup of these patients were studied and scrutinized thoroughly. *Results*. Thirty-four patients were operated while 2 refused surgery. Most of our patients were between 16 and 30 years (55.6%) of age. The commonest presenting complaints were penile swelling and detumescence during sexual intercourse or an erection. All except two of our patients were managed with immediate surgical repair which had excellent results even in the presence of associated urethral injury. *Conclusion*. Fracture of the penis is a surgical emergency which can be best managed by immediate surgical repair with excellent results even in the presence of urethral injury.

## 1. Introduction

Fracture of the penis is a tear in the tunica albuginea of the corpora cavernosa which may be associated with injury to the corpus spongiosum and urethra. Although fracture of the penis can be easily recognised clinically, it is under-reported due to the embarrassing nature of the injury to the patient. A crackling sound, pain, detumescence, bruising, swelling, and bleeding per urethra are the common symptoms reported by the patients. Due to the typical symptoms of fracture of the penis, surgical exploration can be performed without delay, avoiding the need of further diagnostic procedures [1, 2]. None the less if the cause is atypical or obscure, further diagnostic methods should be used to make the diagnosis. In order to avoid complications of conservative management, such as chordee or failure to attain erection, urgent and immediate surgical exploration is mandatory [3]. Our study was conducted retrospectively with the aim to ascertain the clinical presentation, commonest age group affected, and treatment of patients diagnosed to have penis fracture.

## 2. Materials and Methods

A retrospective study was carried out at Government Medical College & Hospital Srinagar, that is, a tertiary care referral centre in Jammu and Kashmir, over a period of six years from January 2006 to January 2011. All the patients diagnosed to have penile fracture were enrolled in the study group. The diagnosis was made based on the clinical findings in the patients, and no invasive diagnostic procedures were carried out. All the data pertaining to the presentation, management, and followup of these patients were studied and scrutinized thoroughly. All the patients except two, who refused surgery, were operated on the day of presentation to the hospital emergency services. A standard surgical approach, that is, a subcoronal degloving incision in the penile skin, a careful examination of the tunica, corpora, and the urethra to record the extent of the injury followed by evacuation of the hematoma, careful hemostasis, and repair of the tear with slowly absorbing sutures was done. The patients were discharged from the hospital with the advice and medication to suppress erection for one week and abstain from sexual activity for 6–8 weeks. The patients were followed up at one, three, and six months after being discharged from the hospital. The study was approved by the local ethics committee and the institutional review board [GMC-201798].

## 3. Results

During the study period, 36 patients having a fracture of the penis were managed at our hospital. Out of these, 34 patients underwent surgical intervention. Two patients were managed conservatively as they refused surgery. The youngest patient in our study was a 16-year-old boy and the eldest a 67-year-old man. As seen in Table 1, 20 (55.6%) of the patients were less than 30 years of age, and the mean age of presentation was 32.3 years. Twenty-six (72.2%) of our patients were married, and 10 (27.8%) were unmarried.

The time elapsed from sustaining the trauma to presentation at the hospital ranged from 2 hours to 7 days. Twenty-three (63.9%) and 12 (33.3%) patients presented to the hospital within 24 hours and from 24 to 72 hours after sustaining the trauma, respectively. One of the patients presented after 7 days. 

**Figure 1:**
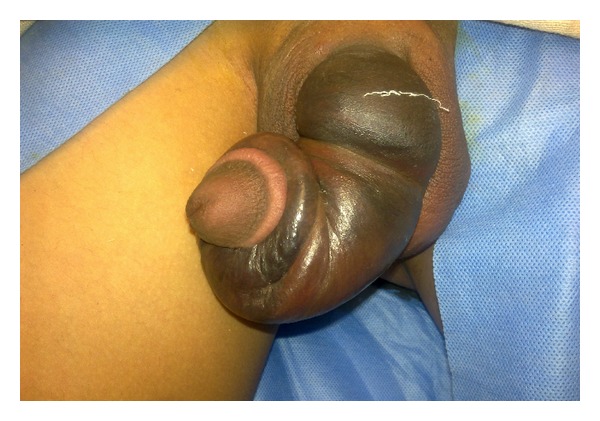
The classical presentation of penis fracture. The hematoma and the bending of penis seen in penile fracture.

As shown in Table 2, the most common presenting complaints were rapid detumescence during erection and penile swelling (Figure 1). Vigorous sexual intercourse was reported by 24 (66.7%) patients as the cause of their injury. Ten (27.8%) patients reported manipulation of the erect penis while masturbating, and 2 (5.5%) reported rolling over an erect penis while sleeping as the causes of their injury.

Twenty-four (66.7%) patients had tears in the right corpora while 10 (27.8%) patients had tears in the left corpora. Moreover, four patients had associated urethral injury (11.11%), that is, small (<0.5 cms) lateral tears of the urethra beneath the cavernosal tears which were managed by pericatheteric primary repair. All the study subjects had unilateral corporal tears ranging from 0.3 to 2 cms in size. All of our patients were discharged within 7 days of their day of admission.

As shown in Table 3, the most common complications noted in the immediate postoperative period and subsequent follow-up visits, in order of decreasing frequency, were permanent induration (16.7%), penile curvature (8.3%), penile skin necrosis (5.6%), and erectile dysfunction (2.8%) in 6, 3, 2, and 1 patients, respectively. According to the patients and their partners the penile curvature was apparently not interfering with sexual intercourse. Penile skin necrosis was managed conservatively and resolved subsequently. The indurations at the sites of repair had no effects on the lifestyles of the patients as reported by them. One of the patients who refused surgery had an erectile dysfunction which was confirmed by carrying out further diagnostic tests; the other one did not return for followup.

## 4. Discussion

Fracture of the penis is a relatively uncommon type of genitourinary trauma, but lately it is being reported increasingly. Tunica albuginea ruptures due to its marked thinning (0.25–0.5 mm during erection from a resting thickness of 2 mm) during erection along with simultaneous marked short-term pressure increase which approaches or exceeds the tensile strength of the tunica during acute abrupt loading or bending of the erect penis [4]. Trauma sustained during sexual intercourse is reported as the main cause of penile injury in United States of America; manipulating the erect penis to achieve detumescence is reported as a major cause in the Middle East [5], whereas rolling over an erect penis in bed and masturbation are the commonest causes in Japan [6]. In our group, vigorous sexual intercourse (66.7%) was the commonest cause followed by manipulation of an erect penis (27.8%) and rolling over an erect penis in bed (5.5%). Most of the patients in our study group were young adults, less than 30 years of age (55.6%), in concordance with earlier studies [7–9]. Ghilan et al. [7] reported 56.7% of their patients to be unmarried, attributing this to the fact that their patients were living in a conservative community and had sustained the trauma due to hard penile manipulation while masturbating, as it was the easiest available option to attain sexual relief whereas 26 (72.2%) of our patients were married during the study period.

Most of the times only the patients history and physical examination are all that is needed to make the correct diagnosis. A physician can easily recognise fracture of the penis as it has pathognomic clinical symptoms. An audible sudden cracking or snapping sound heard by the patient himself is the commonest presenting complaint followed by sudden rapid detumescence, pain, swelling, ecchymosis, and deformation of the penis [10, 11]. Urethral injury is to be suspected if there is history or presence of blood at the external urethral meatus, gross hematuria, or inability to pass urine, and these should be further investigated by retrograde urethrography [12, 13]. In obscure or atypical cases, additional investigative modalities such as echotomography, Doppler ultrasonography, and magnetic resonance imaging help in making the diagnosis [14–16]. Our study revealed that the most common presenting complaints were penile detumescence during sexual activity and penile swelling (100%), followed by crackling sound (97.2%), pain (94.5%), and bleeding per urethra (5.6%).

Treatment of the penile fracture has been a controversial issue [17, 18]. Earlier reports usually recommended nonsurgical management, that is, conservative, which included bed rest, pressure dressings, catheterization, and ice packs for 24–48 hours in addition to antibiotics, fibrinolytics, oestrogens, and diazepam for suppressing erection [19, 20]. Ten to thirty percent of patients receiving such conservative management developed impaired erections, permanent deformity, or suboptimal coitus [1]. Kalash and Young Jr. [17] reported that the complication rate of conservative treatment before and after 1971 was 10 percent and 53 percent, respectively, including deformity of the penis, pulsatile diverticulum, decrease in rigidity, and failure of conservative treatment. Early surgical treatment was strongly recommended by the authors because of the excellent results, shorter hospitalization, less morbidity, and early return to full sexual activity. Their review also revealed that the incidence of associated urethral injury before and after 1971 was 33 percent and 14 percent, respectively. Hinev [11] in his review has recommended immediate surgical treatment of all cases of penile fracture; also emergency surgical repair offers a chance for complete recovery, even in the presence of urethral injury and is the best method for providing a good functional prognosis. It has been shown that urethral injuries can be closed in a spatulated, watertight fashion with subsequent urethral catheter drainage for at least three weeks [21]. Patients should be counselled to abstain from sexual activity for a period of at least 6–8 weeks [22].

## 5. Conclusion

Fracture of the penis is a surgical emergency which can be best managed by immediate surgical repair. No further investigations other than a thorough history and clinical examination are needed for making a diagnosis of penile fracture most of the times. Immediate surgical repair has the best prognosis even in the presence of urethral injury.

## Figures and Tables

**Table 1:** Age distribution.

Age group (years)	Number of patients	%
16–30	20	55.6
31–45	13	36.1
>45	3	8.3

**Table 2:** Presenting complaints.

Presenting complaints	Number of patients	%
Detumescence	36	100
Penile swelling	36	100
Crackling sound	35	97.2
Pain	34	94.4
Bleeding per urethra	2	5.6

**Table 3:** Complications.

Complication	Number of patients	%
Permanent induration	6	16.7
Penile curvature	3	8.3
Penile skin necrosis	2	5.6
Erectile dysfunction	1	2.8
